# Supplementary material for: Immune cell profiling of the cerebrospinal fluid enables the characterization of the brain metastasis microenvironment
Source: Nat Commun. 2021 Mar 8;12:1503. doi: 10.1038/s41467-021-21789-x (PMC7940606; doi:10.1038/s41467-021-21789-x)
Supplement: Supplementary file 1 — Supplementary Information [file 41467_2021_21789_MOESM1_ESM.pdf]

SUPPLEMENTARY INFORMATION

Supplementary figure 1, associated with Figure 1. Analysis of tumor immune infiltration by targeted gene expression and IHC. **3**

Supplementary figure 2, associated with Figure 2. scRNAseq data processing in the tumor cohort. **5**

Supplementary figure 3, associated with Figure 2. scRNAseq broad cell type annotation in the tumor cohort. **7**

Supplementary figure 4, associated with Figure 2. scRNAseq TAM and microglia cell type annotation in the tumor cohort. **9**

Supplementary figure 5, associated with Figure 2. scRNAseq T cell & NK cell type annotation in the tumor cohort **11**

Supplementary figure 6, associated with Figure 2. scRNAseq proliferating leukocytes annotation in the tumor cohort **13**

Supplementary figure 7, associated with Figure 3. single-cell RNA CSF immune cell type identification in the matched tumor-CSF sample cohort. **15**

Supplementary figure 8, associated with Figure 3. Flow cytometry immune cell type identification through the general leukocyte panel, in the matched tumor-CSF sample cohort. **17**

Supplementary figure 9, associated with Figure 3. Flow cytometry immune cell type identification through the T cell panel, in the matched tumor-CSF sample cohort. **19**

Supplementary figure 10, associated with Figure 4. T cell TCR profiling across BrM. **21**

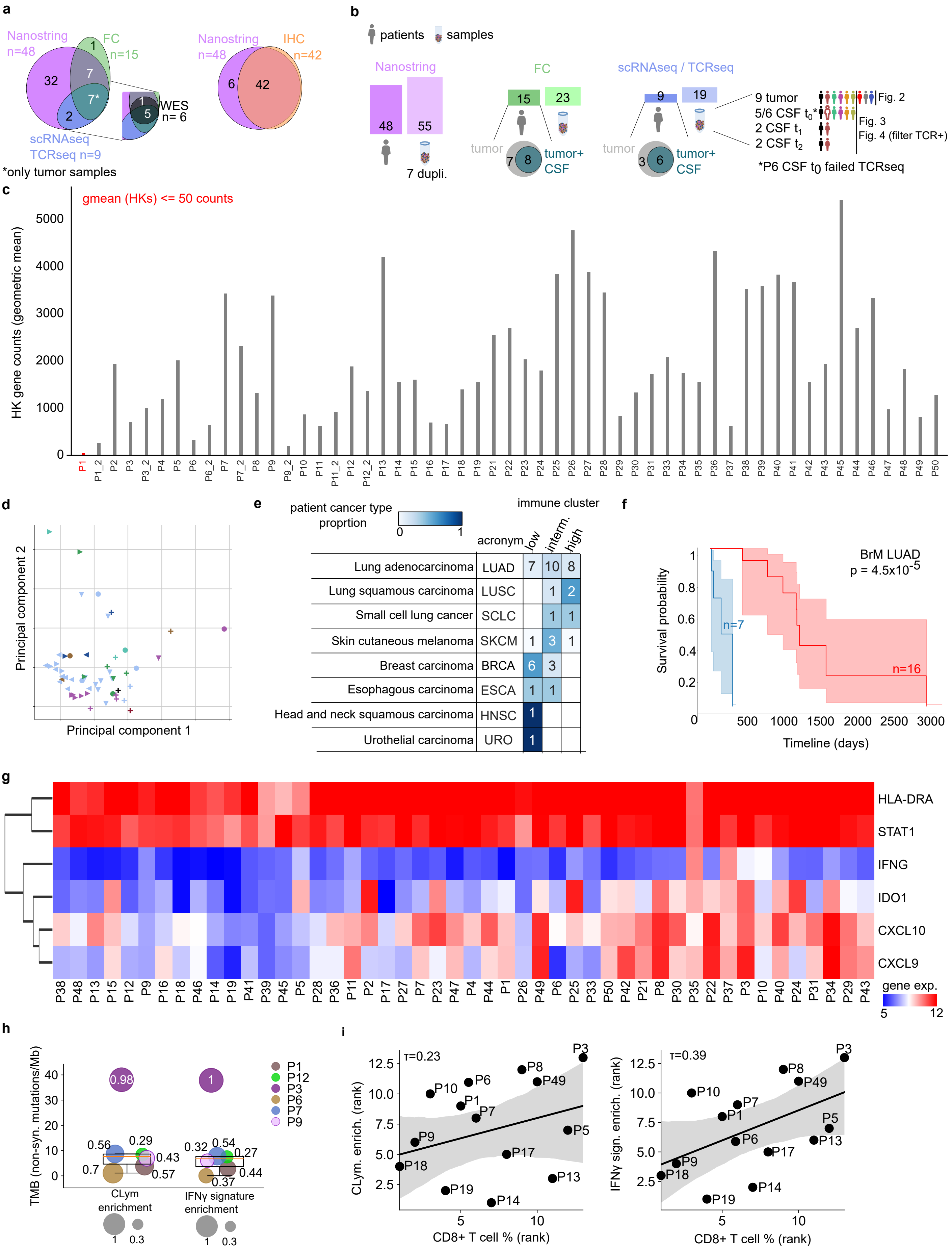
a
Nanostring n=48
FC n=15
32
1
7
7*
2
scRNAseq TCRseq n=9
1
5
WES n= 6
Nanostring n=48
IHC n=42
6
42
*only tumor samples
b
patients
samples
Nanostring
48
55
7 dupli.
FC
15
23
tumor
7
8
tumor+ CSF
scRNAseq / TCRseq
9
19
tumor
3
6
tumor+ CSF
9 tumor
5/6 CSF t_0*
2 CSF t_1
2 CSF t_2
Fig. 2
Fig. 3
Fig. 4 (filter TCR+)
*P6 CSF t_0 failed TCRseq
c
gmean (HKs) <= 50 counts
HK gene counts (geometric mean)
P1 P1_2 P2 P3 P3_2 P4 P5 P6 P6_2 P7 P7_2 P8 P9 P9_2 P10 P11 P11_2 P12 P12_2 P13 P14 P15 P16 P17 P18 P19 P21 P22 P23 P24 P25 P26 P27 P28 P29 P30 P31 P33 P34 P35 P36 P37 P38 P39 P40 P41 P42 P43 P45 P44 P46 P47 P48 P49 P50
d
Principal component 2
Principal component 1
e
patient cancer type proprtion
0
1
immune cluster
acronym
low
interm.
high
Lung adenocarcinoma | LUAD | 7 | 10 | 8
Lung squamous carcinoma | LUSC | | 1 | 2
Small cell lung cancer | SCLC | | 1 | 1
Skin cutaneous melanoma | SKCM | 1 | 3 | 1
Breast carcinoma | BRCA | 6 | 3 |
Esophagous carcinoma | ESCA | 1 | 1 |
Head and neck squamous carcinoma | HNSC | 1 | |
Urothelial carcinoma | URO | 1 | |
f
BrM LUAD
p = 4.5x10^{-5}
Survival probability
n=7
n=16
Timeline (days)
g
HLA-DRA
STAT1
IFNG
IDO1
CXCL10
CXCL9
P38 P48 P13 P15 P12 P9 P16 P18 P46 P14 P19 P41 P39 P45 P5 P28 P36 P11 P2 P17 P27 P7 P23 P47 P4 P44 P1 P26 P49 P6 P25 P33 P50 P42 P21 P8 P30 P35 P22 P37 P3 P10 P40 P24 P31 P34 P29 P43
gene exp.
5
12
h
TMB (non-syn. mutations/Mb)
0.98
1
0.56
0.29
0.43
0.7
0.57
0.32
0.54
0.27
0.44
0.37
CLym enrichment
IFNγ signature enrichment
1
0.3
P1
P12
P3
P6
P7
P9
i
τ=0.23
CLym. enrich. (rank)
CD8+ T cell % (rank)
P3 P8 P6 P49 P10 P1 P7 P5 P9 P17 P18 P13 P19 P14
τ=0.39
IFNγ sign. enrich. (rank)
CD8+ T cell % (rank)
P3 P8 P49 P10 P7 P1 P5 P6 P13 P17 P9 P18 P14 P19

Supplementary figure 1, associated with Figure 1. Analysis of tumor immune infiltration by targeted gene expression and IHC.

a) Venn diagram showing the patients analyzed across the distinct techniques: Nanostring RNA profiling, Flow Cytometry (FC), Whole Exome Sequencing (WES), Immunohistochemistry (IHC), single cell-RNA (scRNA) and TCR sequencing. **b)** Schematic representation of the patient-sample usage per experimental technique, same experimental techniques as in a) are shown. **c)** Bar plot representing the average geometric mean of Housekeeping (HK) gene counts per sample according to targeted gene expression experiments (Nanostring PanCancer Immune panel v1). Note that duplicate samples have been included, labeled as Px_2 (see sample quality selection criteria in Methods). **d)** Principal component analysis of Nanostring PanCancer Immune panel normalised counts per sample. Each marker represents a sample, markers have been colored according to the primary tumor of origin and shaped according to distinct runs (**Supp. Data 3**). See how there is no clustering by run, evicting the absence of batch effect. **e)** Heatmap representing the distribution of samples per immune cluster according to the primary tumor of origin (see Fig. 1c). Cancer type full names have been included. **f)** Kapplan-Meier survival estimation for BrM patients with primary tumor lung adenocarcinoma (LUAD) stratified based on the immune cluster (low vs intermediate+high). Statistical significance according to the log rank test is shown. The number of patients included in each group (n) is shown. **g)** Heatmap representing the expression values of the six genes integrating the *refined* IFNɣ signature (Ayers et al 2017) (rows) in 48 BrM patients (columns). Columns have been sorted as in Fig. 1c. On the left, the dendrogram of gene aggregation, according to expression values of the six genes across samples, is shown. **h)** Box plot representing the tumor mutational burden in six patients where WES was performed. Each dot represents a patient, each colored distinctively, and the size the enrichment of cytotoxic lymphocytes and IFNɣ signature. All boxplots indicate median (center line), 25th and 75th percentiles (bounds of box), and minimum and maximum (whiskers) **i)** Regression plots representing the Spearman correlation between cytotoxic lymphocytes (left) and IFNɣ signature (right) enrichment score and percentage of $CD8^+$ T cells detected by FC (measured as $CD3^+$ $CD8^+$ cells inside the total population of alive cells). Each dot represents a unique patient sample, Spearman test results are shown (τ and p-value as an asterisk if < 0.05). C.I. by performing a multilevel bootstrap, that resamples both units and observations (within unit), have also been displayed as shaded areas.

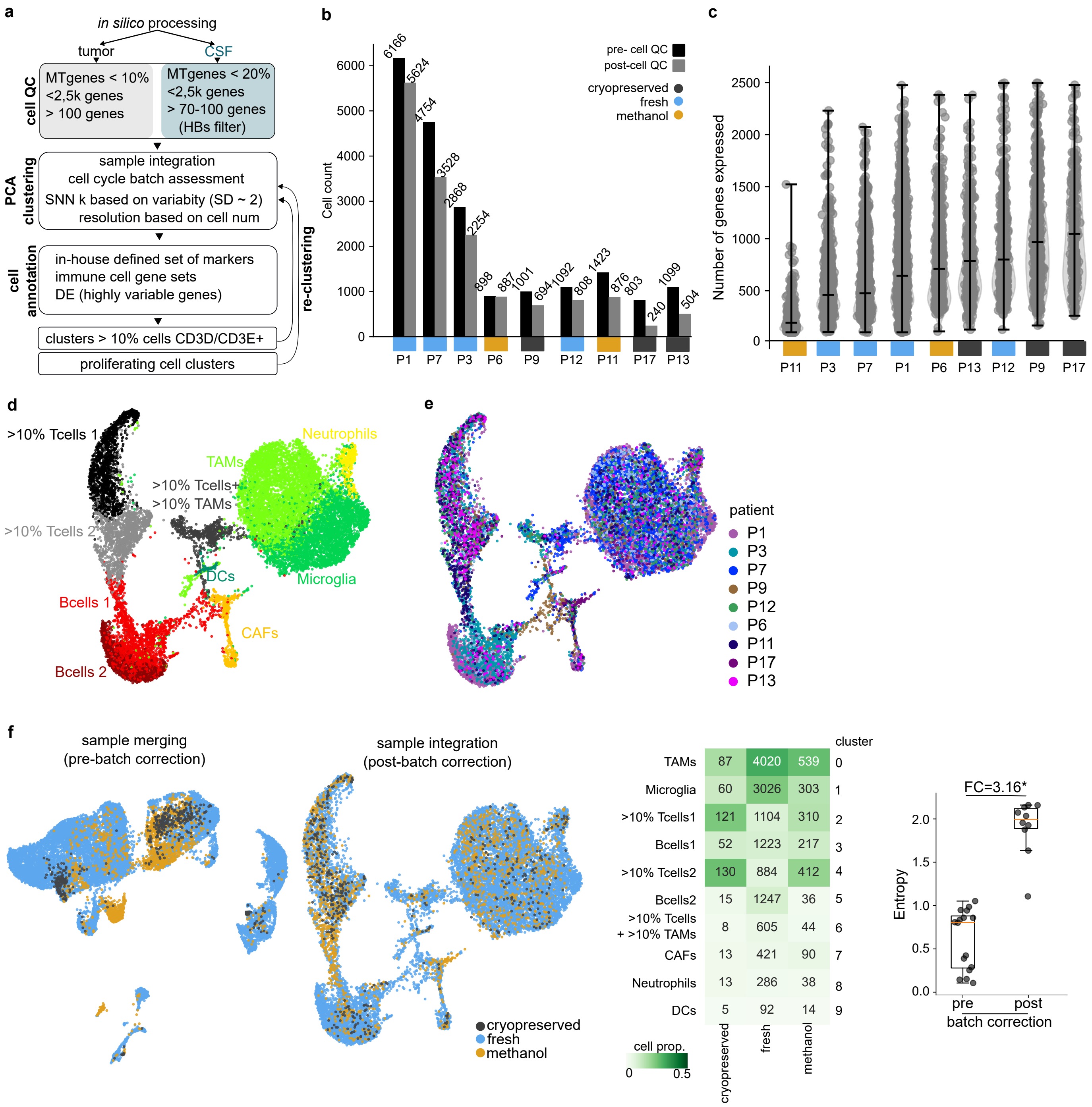
a
in silico processing
tumor
CSF
cell QC
MTgenes < 10%
<2,5k genes
> 100 genes
MTgenes < 20%
<2,5k genes
> 70-100 genes
(HBs filter)
PCA clustering
sample integration
cell cycle batch assessment
SNN k based on variability (SD ~ 2)
resolution based on cell num
cell annotation
in-house defined set of markers
immune cell gene sets
DE (highly variable genes)
clusters > 10% cells CD3D/CD3E+
proliferating cell clusters
re-clustering
b
Cell count
pre- cell QC
post-cell QC
cryopreserved
fresh
methanol
6166 5624 4754 3528 2868 2254 898 887 1001 694 1092 808 1423 876 803 240 1099 504
P1 P7 P3 P6 P9 P12 P11 P17 P13
c
Number of genes expressed
P11 P3 P7 P1 P6 P13 P12 P9 P17
d
>10% Tcells 1
Neutrophils
TAMs
>10% Tcells+
>10% TAMs
>10% Tcells 2
DCs
Microglia
Bcells 1
CAFs
Bcells 2
e
patient
P1
P3
P7
P9
P12
P6
P11
P17
P13
f
sample merging
(pre-batch correction)
sample integration
(post-batch correction)
cryopreserved
fresh
methanol
cluster
TAMs 87 4020 539 0
Microglia 60 3026 303 1
>10% Tcells1 121 1104 310 2
Bcells1 52 1223 217 3
>10% Tcells2 130 884 412 4
Bcells2 15 1247 36 5
>10% Tcells + >10% TAMs 8 605 44 6
CAFs 13 421 90 7
Neutrophils 13 286 38 8
DCs 5 92 14 9
cryopreserved
fresh
methanol
cell prop.
0
0.5
FC=3.16*
Entropy
pre
post
batch correction

Supplementary figure 2, associated with Figure 2. scRNAseq data processing in the tumor cohort.

a) Diagram representing the *in silico* methodology followed for scRNAseq cell type annotation. **b)** Paired bar plot representing the number of infiltrating cells sequenced for each patient (n=9 patients) (black) and the number of cells remaining for analysis after cell quality filtering, as specified in a) (grey). A colored annotation on the sample preservation technique is shown. It can be observed how even if fresh samples tended to have a larger number of cells, one of them (P12) was in the cell number range of methanol and cryopreserved samples. **c)** Violin plot representing the distribution of the number of detected genes per cell across tumor samples, as a measure of RNA quality. All violin plots indicate median (center line), 25th and 75th percentiles (bounds of box), and minimum and maximum (whiskers). Each dot represents a cell. The total of cells in each boxplot can be found in b) post-cell QC number. A colored annotation on the sample preservation technique is shown. It can be observed how there is no clear pattern of RNA integrity by preservation technique. Thus, given the tumor sample cohort analyzed, all preservation techniques seem to have a similar degree of RNA integrity. **d)** UMAP projection of the cell clusters identified by integrating 9 tumor samples together and colored by the general cell cluster initial annotations, prior to re-clustering. **e)** UMAP projection in (d) colored by patient ID. It shows how there is no batch effect by patient sample. **f)** Left, UMAP projection pre- and post- batch adjustment through sample *integration* Seurat method (see Methods), colored by sample preservation technology. Middle panel displays a heatmap showing the number of cells per cell cluster across sample preservation technique, post- batch adjustment. Right, boxplot representing the entropy score of the cell distribution across sample techniques per each cell cluster. Each dot represents a cell cluster; the total of analysed clusters are the ones shown in the middle panel heatmap (n=10). Same parameters (dimensions and granularity) have been used to identify the cell clusters in merged (pre-batch correction) vs integrated (post-batch correction) sample cohorts. It can be observed how the entropy is significantly higher after batch correction (represented as an asterisk, Mann Whitney U test p-value < 0.05) meaning that cells are more equally distributed across sample preservation techniques in each cell cluster; implying that batch effect can be properly adjusted. All boxplots indicate median (center line), 25th and 75th percentiles (bounds of box), and minimum and maximum (whiskers).

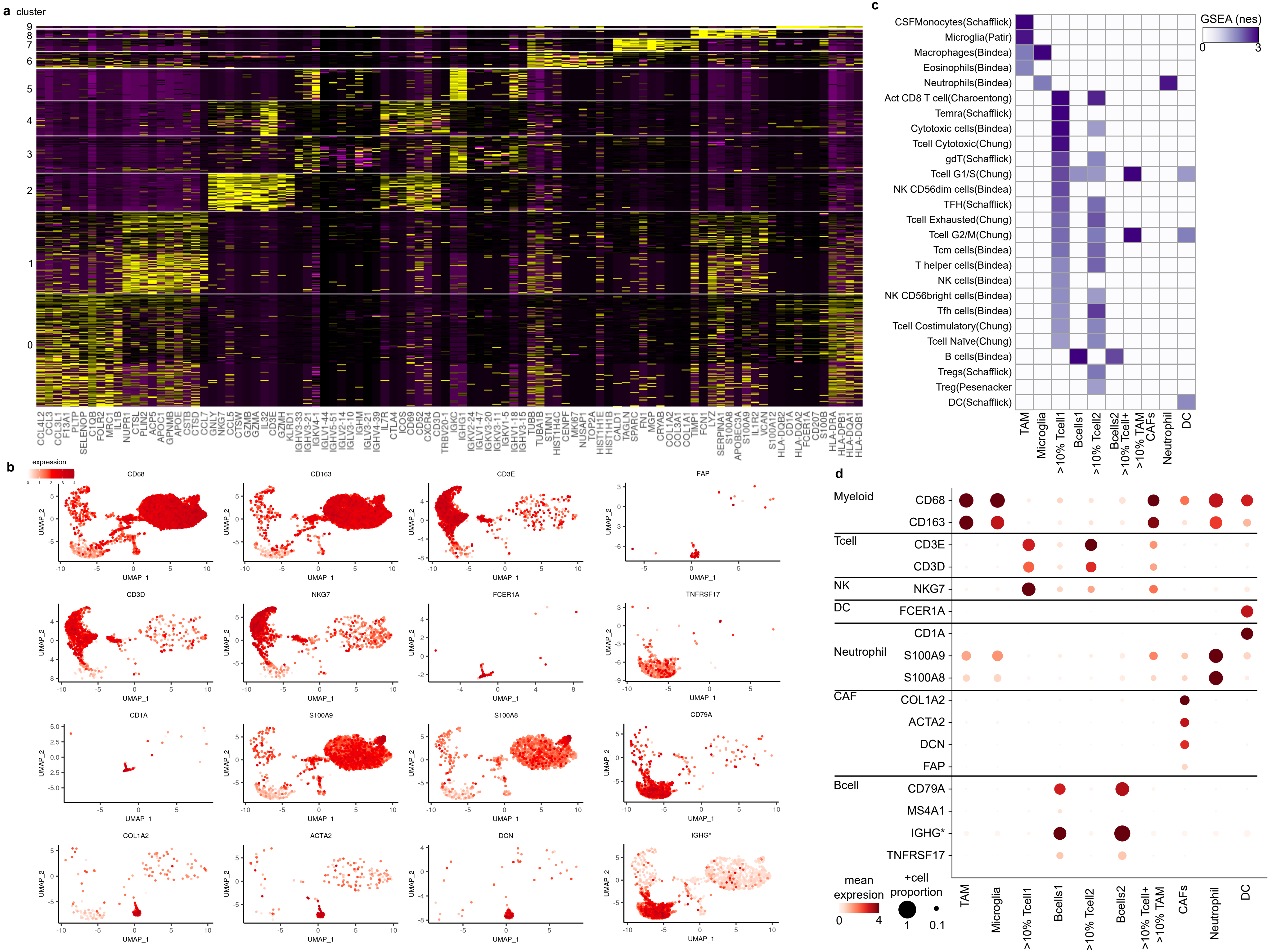
a cluster
9
8
7
6
5
4
3
2
1
0
CCL4L2 CCL3 CCL3L1 F13A1 PLTP SELENOP C1QB FOLR2 MRC1 IL1B NUPR1 CTSL PLIN2 ACP5 APOC1 GPNMB APOE CSTB CSTD CCL7 GNLY NKG7 CCL5 CTSW GZMB GZMA IL32 CD3E GZMH KLRD1 IGHV3-33 IGHV3-21 IGKV4-1 IGLV1-44 IGHV5-51 IGLV2-14 IGLV3-10 IGHM IGLV3-21 IGHV4-39 IL7R CTLA4 ICOS CD69 CD52 CXCR4 CD3D TRBV20-1 IGKC IGHG1 IGKV2-24 IGLV1-47 IGKV3-20 IGKV3-11 IGKV1-5 IGHV1-18 IGHV3-15 TUBB TUBA1B STMN1 HIST1H4C CENPF MKI67 NUSAP1 TOP2A HIST1H1B HIST1H1E CALD1 TAGLN SPARC FN1 MGP CRYAB COL1A2 COL3A1 COL1A1 TIMP1 FCN1 LYZ SERPINA1 S100A8 APOBEC3A S100A9 IL1R2 VCAN S100A12 HLA-DQB2 CD1A HLA-DQA2 FCER1A CD207 S100B HLA-DRA HLA-DPB1 HLA-DQA1 HLA-DQB1
b
expression
CD68
CD163
CD3E
FAP
CD3D
NKG7
FCER1A
TNFRSF17
CD1A
S100A9
S100A8
CD79A
COL1A2
ACTA2
DCN
IGHG*
UMAP_1
UMAP_2
c
GSEA (nes)
0
3
CSFMonocytes(Schafflick)
Microglia(Patir)
Macrophages(Bindea)
Eosinophils(Bindea)
Neutrophils(Bindea)
Act CD8 T cell(Charoentong)
Temra(Schafflick)
Cytotoxic cells(Bindea)
Tcell Cytotoxic(Chung)
gdT(Schafflick)
Tcell G1/S(Chung)
NK CD56dim cells(Bindea)
TFH(Schafflick)
Tcell Exhausted(Chung)
Tcell G2/M(Chung)
Tcm cells(Bindea)
T helper cells(Bindea)
NK cells(Bindea)
NK CD56bright cells(Bindea)
Tfh cells(Bindea)
Tcell Costimulatory(Chung)
Tcell Naïve(Chung)
B cells(Bindea)
Tregs(Schafflick)
Treg(Pesenacker
DC(Schafflick)
TAM
Microglia
>10% Tcell1
Bcells1
>10% Tcell2
Bcells2
>10% Tcell+ >10% TAM
CAFs
Neutrophil
DC
d
Myeloid
CD68
CD163
Tcell
CD3E
CD3D
NK
NKG7
DC
FCER1A
CD1A
Neutrophil
S100A9
S100A8
CAF
COL1A2
ACTA2
DCN
FAP
Bcell
CD79A
MS4A1
IGHG*
TNFRSF17
mean expresion
0
4
+cell proportion
1
0.1
TAM
Microglia
>10% Tcell1
Bcells1
>10% Tcell2
Bcells2
>10% Tcell+ >10% TAM
CAFs
Neutrophil
DC

Supplementary figure 3, associated with Figure 2. scRNAseq broad cell type annotation in the tumor cohort.

a) Heatmap representing the top genes (columns) differentially expressed (DE) across cell clusters (rows), based on Seurat gene markers analysis. Heatmap cells represent the average gene expression (Z-scaled from yellow -2 to violet +2) in the cell cluster. See cluster cell annotation in (**Supp. Fig 2f**). **b)** UMAP projection in **Supp. Fig. 2d** and **Fig. 2a** colored by the expression (in a red color scale) of reference broad cell lineage gene markers. **c)** Heatmap displaying the results of Gene Set Enrichment Analysis (GSEA, normalised enrichment score, NES) of DE genes in high quality immune gene sets gathered from the literature. Only enrichments with an FDR < 0.25 are shown. **d)** Dot plot representing the summary of the expression of reference broad cell lineage gene markers across cell clusters. Dot color represents the average gene expression in the cell cluster and dot size the proportion of cells expressing the marker (UMI > 0) in the cell cluster.

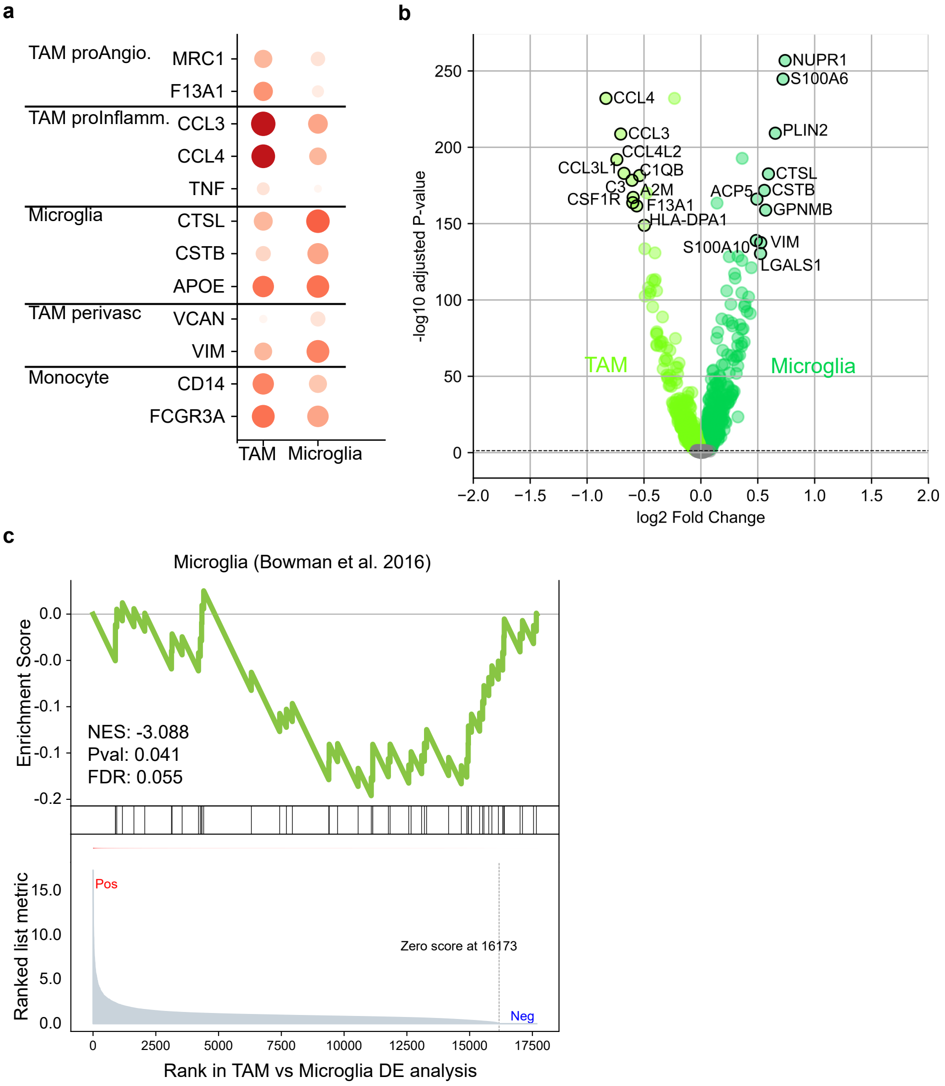
a
TAM proAngio.
MRC1
F13A1
TAM proInflamm.
CCL3
CCL4
TNF
Microglia
CTSL
CSTB
APOE
TAM perivasc
VCAN
VIM
Monocyte
CD14
FCGR3A
TAM
Microglia
b
-log10 adjusted P-value
log2 Fold Change
NUPR1
S100A6
CCL4
CCL3
PLIN2
CCL4L2
CCL3L1
C1QB
CTSL
C3
A2M
ACP5
CSTB
CSF1R
F13A1
GPNMB
HLA-DPA1
S100A10
VIM
LGALS1
TAM
Microglia
c
Microglia (Bowman et al. 2016)
Enrichment Score
NES: -3.088
Pval: 0.041
FDR: 0.055
Ranked list metric
Pos
Zero score at 16173
Neg
Rank in TAM vs Microglia DE analysis

Supplementary figure 4, associated with Figure 2. scRNAseq TAM and microglia cell type annotation in the tumor cohort.

a) Dot plot representing the expression of reference TAM/microglia lineage gene markers across cell clusters. Dot color represents the average gene expression in the cell cluster and dot size the proportion of cells expressing the marker (UMI > 0) in the cell cluster. **b)** Volcano plot of the genes DE between cell clusters annotated as TAM and microglia. Top-10 DE genes in TAM and microglia are shown. X-axis represents the $\log_2$ fold change and Y-axis the -log10 adjusted (FDR) p-value according to Mann Whitney U test. **c)** GSEA of the DE genes between TAM and microglia in Bowman et al (2016) reference macrophage and microglia gene sets. We found an up-regulation of the TAM gene set (data not shown; FDR > 0.25) and a significant (FDR < 0.25) down-regulation of the Microglia gene set, the enrichment plot produced by GSEA is shown.

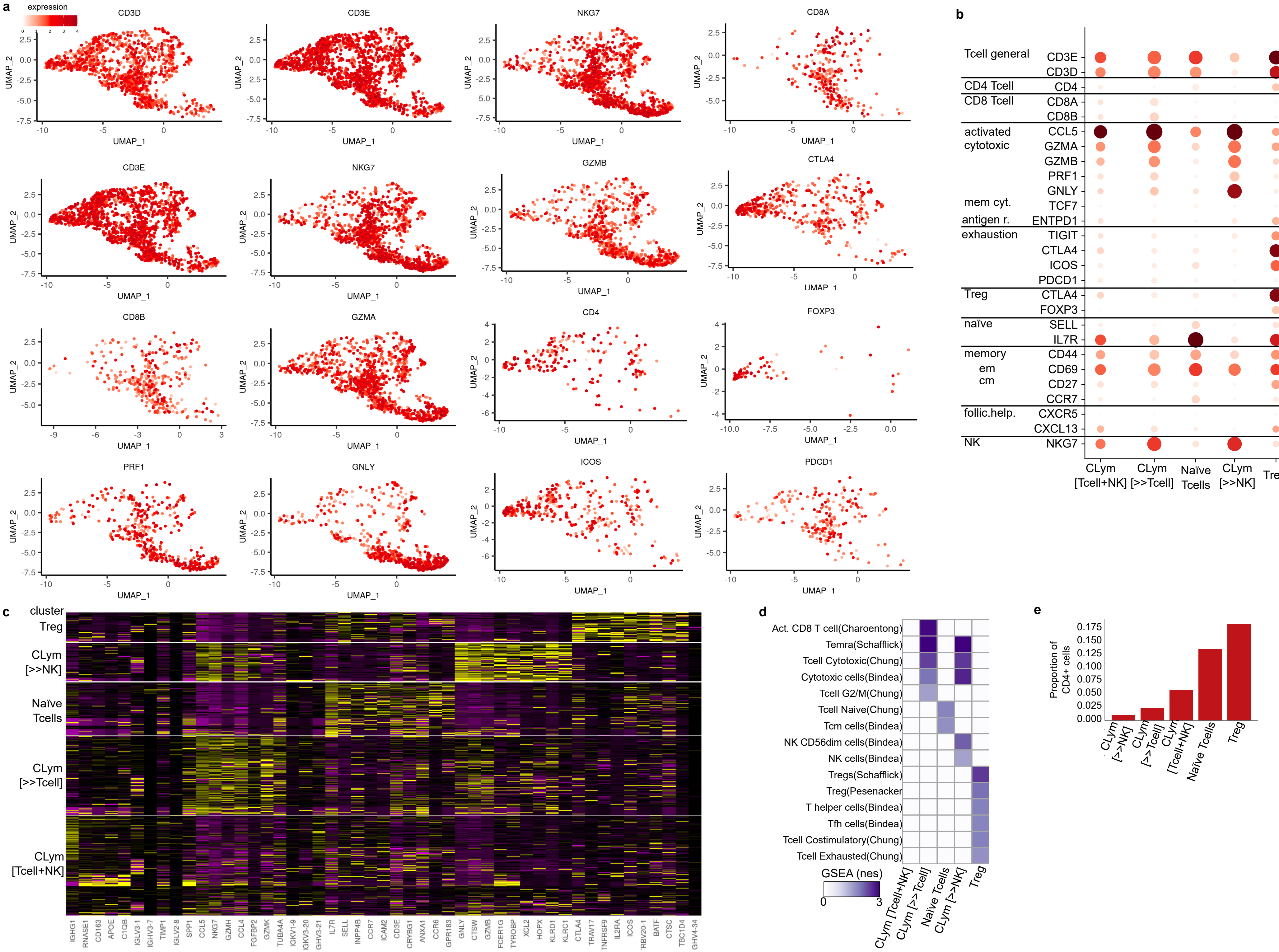

a
expression
CD3D
CD3E
NKG7
CD8A
GZMB
CTLA4
CD8B
GZMA
CD4
FOXP3
PRF1
GNLY
ICOS
PDCD1
UMAP_1
UMAP_2
b
Tcell general
CD3E
CD3D
CD4 Tcell
CD4
CD8 Tcell
CD8A
CD8B
activated cytotoxic
CCL5
GZMA
GZMB
PRF1
GNLY
mem cyt.
TCF7
antigen r.
ENTPD1
exhaustion
TIGIT
CTLA4
ICOS
PDCD1
Treg
CTLA4
FOXP3
naïve
SELL
IL7R
memory
em
cm
CD44
CD69
CD27
CCR7
follic.help.
CXCR5
CXCL13
NK
NKG7
CLym [Tcell+NK]
CLym [>>Tcell]
Naïve Tcells
CLym [>>NK]
Treg
c
cluster
Treg
CLym [>>NK]
Naïve Tcells
CLym [>>Tcell]
CLym [Tcell+NK]
IGHG1
RNASE1
CD163
APOE
C1QB
IGLV3-1
IGHV3-7
TIMP1
IGLV2-8
SPP1
CCL5
NKG7
GZMH
CCL4
FGFBP2
GZMK
TUBA4A
IGKV1-9
IGKV3-20
GHV3-21
IL7R
SELL
INPP4B
CCR7
ICAM2
CD3E
CRYBG1
ANXA1
CCR6
GPR183
GNLY
CTSW
GZMB
FCER1G
TYROBP
XCL2
HOPX
KLRD1
KLRC1
CTLA4
TRAV17
TNFRSF9
IL2RA
ICOS
TRBV20-1
BATF
CTSC
TBC1D4
GHV4-34
d
Act. CD8 T cell(Charoentong)
Temra(Schafflick)
Tcell Cytotoxic(Chung)
Cytotoxic cells(Bindea)
Tcell G2/M(Chung)
Tcell Naive(Chung)
Tcm cells(Bindea)
NK CD56dim cells(Bindea)
NK cells(Bindea)
Tregs(Schafflick)
Treg(Pesenacker
T helper cells(Bindea)
Tfh cells(Bindea)
Tcell Costimulatory(Chung)
Tcell Exhausted(Chung)
GSEA (nes)
0
3
CLym [Tcell+NK]
CLym [>>Tcell]
Naïve Tcells
CLym [>>NK]
Treg
e
Proportion of CD4+ cells
0.175
0.150
0.125
0.100
0.075
0.050
0.025
0.000
CLym [>>NK]
CLym [>>Tcell]
CLym [Tcell+NK]
Naïve Tcells
Treg

Supplementary figure 5, associated with Figure 2. scRNAseq T cell & NK cell type annotation in the tumor cohort

a) UMAP projection of the Tcell and NK reclustering shown in Fig. 2a, colored by the expression (in a red color scale) of reference T and NK cell lineage gene markers. **b)** Dot plot representing the expression of T cell & NK lineage gene markers across cell clusters. Dot color represents the average gene expression in the cell cluster and dot size the proportion of cells expressing the marker (UMI > 0) in the cell cluster. **c)** Heatmap representing the top genes (columns) differentially expressed across T cell & NK cell clusters (rows), based on Seurat gene markers analysis. Heatmap cells represent the average gene expression (Z-scaled from yellow -2 to violet +2) in the cell cluster. **d)** Heatmap displaying the results of GSEA of DE genes from T cell & NK cell clusters in high quality immune gene sets gathered from the literature. Only enrichments with an FDR < 0.25 are shown. **e)** Bar plot representing the proportion of $CD4^+$ cells (UMI > 0) per cell cluster.

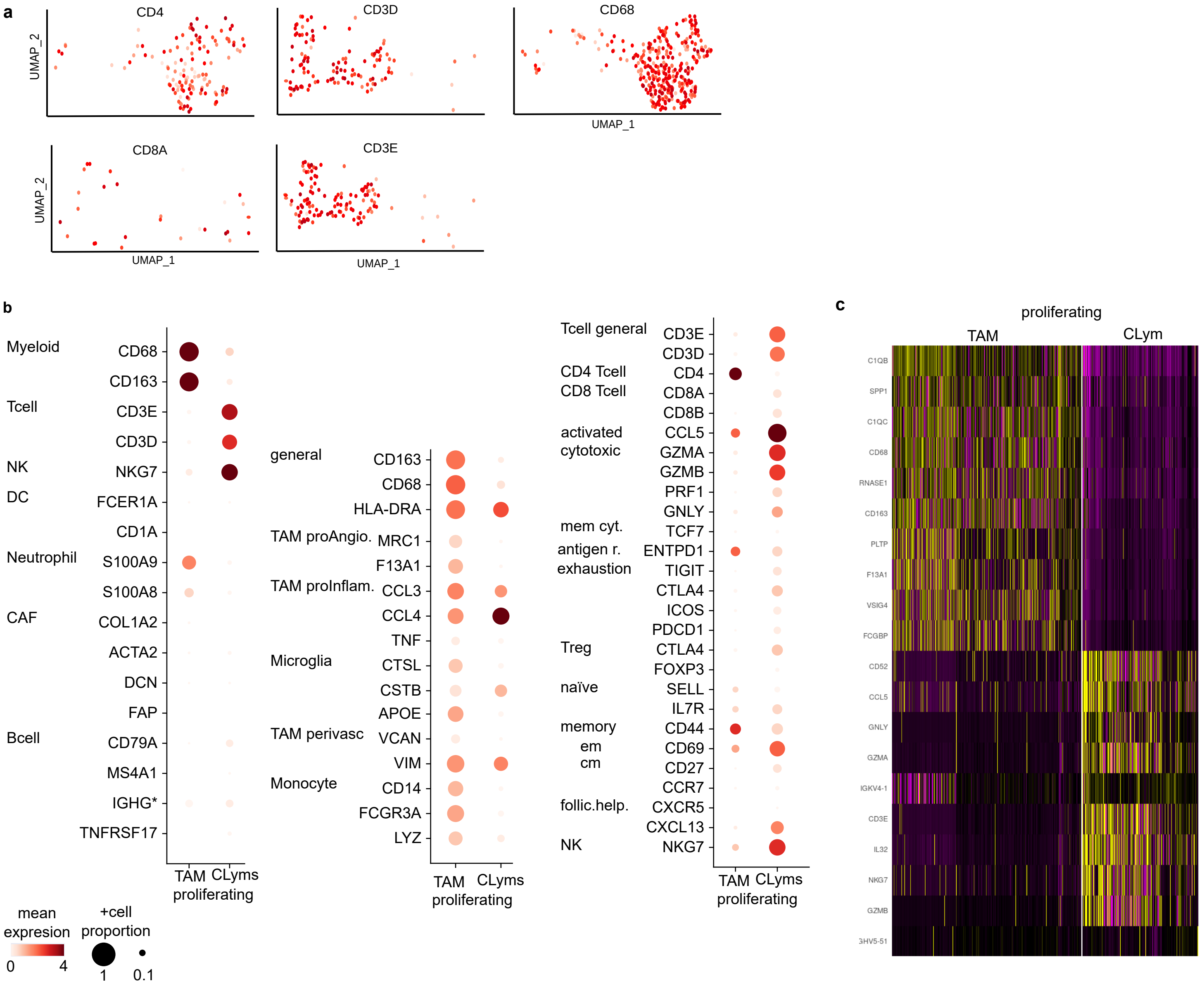
a
CD4
CD3D
CD68
CD8A
CD3E
UMAP_2
UMAP_1
b
Myeloid
Tcell
NK
DC
Neutrophil
CAF
Bcell
CD68
CD163
CD3E
CD3D
NKG7
FCER1A
CD1A
S100A9
S100A8
COL1A2
ACTA2
DCN
FAP
CD79A
MS4A1
IGHG*
TNFRSF17
TAM CLyms
proliferating
general
TAM proAngio.
TAM proInflam.
Microglia
TAM perivasc
Monocyte
CD163
CD68
HLA-DRA
MRC1
F13A1
CCL3
CCL4
TNF
CTSL
CSTB
APOE
VCAN
VIM
CD14
FCGR3A
LYZ
Tcell general
CD4 Tcell
CD8 Tcell
activated
cytotoxic
mem cyt.
antigen r.
exhaustion
Treg
naïve
memory
em
cm
follic.help.
NK
CD3E
CD3D
CD4
CD8A
CD8B
CCL5
GZMA
GZMB
PRF1
GNLY
TCF7
ENTPD1
TIGIT
CTLA4
ICOS
PDCD1
CTLA4
FOXP3
SELL
IL7R
CD44
CD69
CD27
CCR7
CXCR5
CXCL13
NKG7
mean
expresion
0
4
+cell
proportion
1
0.1
c
proliferating
TAM
CLym
C1QB
SPP1
C1QC
CD68
RNASE1
CD163
PLTP
F13A1
VSIG4
FCGBP
CD52
CCL5
GNLY
GZMA
IGKV4-1
CD3E
IL32
NKG7
GZMB

Supplementary figure 6, associated with Figure 2. scRNAseq proliferating leukocytes annotation in the tumor cohort

a) UMAP projection of the proliferating leukocytes reclustering shown in Fig. 2a, colored by the expression (in a red color scale) of reference broad cell lineage gene markers. **b)** Dot plots representing the expression of reference broad cell lineage gene markers (left panel), myeloid lineage markers (middle) and T cell & NK lineage cell markers (right) across proliferating leukocyte cell clusters. Dot color represents the average gene expression in the cell cluster and dot size the proportion of cells expressing the marker (UMI > 0) in the cell cluster. **c)** Heatmap representing the top genes (rows) differentially expressed across proliferating leukocytes clusters (columns), based on Seurat gene markers analysis. Heatmap cells represent the average gene expression (Z-scaled from yellow -2 to violet +2) in the cell cluster.

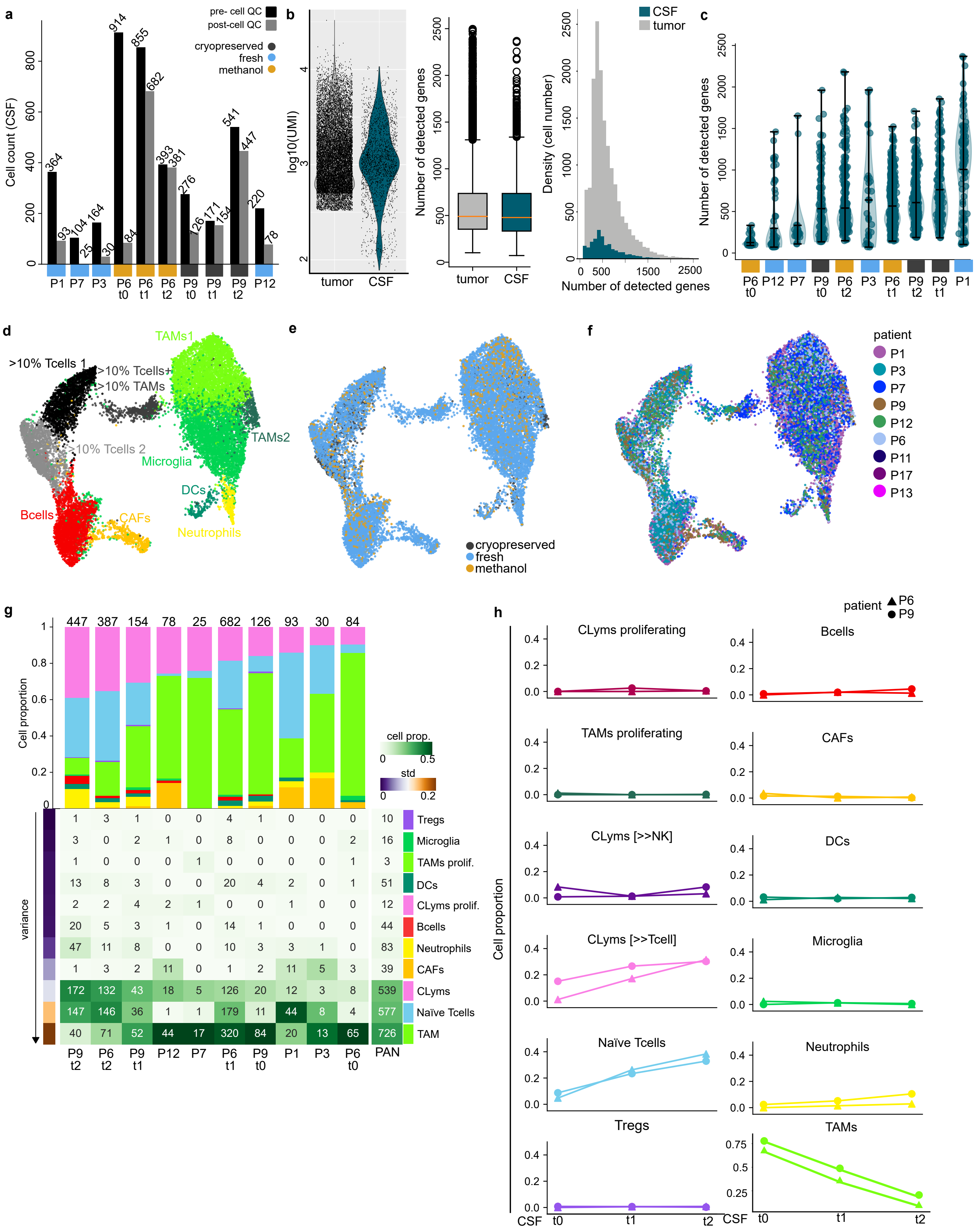
a
pre- cell QC
post-cell QC
cryopreserved
fresh
methanol
Cell count (CSF)
364 93 104 25 164 30 914 84 855 682 393 381 276 126 171 154 541 447 220 78
P1 P7 P3 P6 t0 P6 t1 P6 t2 P9 t0 P9 t1 P9 t2 P12
b
log10(UMI)
tumor CSF
Number of detected genes
tumor CSF
CSF
tumor
Density (cell number)
Number of detected genes
c
Number of detected genes
P6 t0 P12 P7 P9 t0 P6 t2 P3 P6 t1 P9 t2 P9 t1 P1
d
TAMs1
>10% Tcells 1
>10% Tcells+
>10% TAMs
>10% Tcells 2
TAMs2
Microglia
DCs
Bcells
CAFs
Neutrophils
e
cryopreserved
fresh
methanol
f
patient
P1 P3 P7 P9 P12 P6 P11 P17 P13
g
447 387 154 78 25 682 126 93 30 84
Cell proportion
cell prop.
std
variance
1 3 1 0 0 4 1 0 0 0 10 Tregs
3 0 2 1 0 8 0 0 0 2 16 Microglia
1 0 0 0 1 0 0 0 0 1 3 TAMs prolif.
13 8 3 0 0 20 4 2 0 1 51 DCs
2 2 4 2 1 0 0 1 0 0 12 CLyms prolif.
20 5 3 1 0 14 1 0 0 0 44 Bcells
47 11 8 0 0 10 3 3 1 0 83 Neutrophils
1 3 2 11 0 1 2 11 5 3 39 CAFs
172 132 43 18 5 126 20 12 3 8 539 CLyms
147 146 36 1 1 179 11 44 8 4 577 Naïve Tcells
40 71 52 44 17 320 84 20 13 65 726 TAM
P9 t2 P6 t2 P9 t1 P12 P7 P6 t1 P9 t0 P1 P3 P6 t0 PAN
h
patient P6 P9
CLyms proliferating
Bcells
TAMs proliferating
CAFs
CLyms [>>NK]
DCs
CLyms [>>Tcell]
Microglia
Naïve Tcells
Neutrophils
Tregs
TAMs
Cell proportion
CSF t0 t1 t2

Supplementary figure 7, associated with Figure 3. single-cell RNA CSF immune cell type identification in the matched tumor-CSF sample cohort.

Panels correspond to the scRNAseq sample cohort (n=6 patients, n=16 samples). **a)** Paired bar plot representing the number of CSF cells sequenced for each patient sample (n=10 samples) (black) and the number of cells remaining for analysis after quality filtering (see **Supp. Fig. 2a**, Methods) (grey). A colored annotation on the sample preservation technique is shown. **b)** Detected UMIs and genes in tumor vs CSF samples. Left panel, violin plot showing the distribution of the UMIs across all cells from tumor vs CSF samples. Middle panel, box plot showing the distribution of the number of detected genes across all cells from tumor vs CSF samples. Right panel, histogram showing the distribution of the number of detected across cells from tumor vs CSF samples. The number of detected genes has been used as a measure of RNA quality, it can be observed how the distribution is similar in the tumor and the CSF, being both sample types comparable. All boxplots indicate median (center line), 25th and 75th percentiles (bounds of box), and minimum and maximum (whiskers). **c)** Violin plot representing the distribution of number of detected genes per cell across CSF samples, as a measure of RNA quality. Each dot represents a cell. All violin plots indicate median (center line), 25th and 75th percentiles (bounds of box), and minimum and maximum (whiskers). A colored annotation on the sample preservation technique is shown. It can be observed how there is no clear pattern of RNA integrity by preservation technique. Thus, given the tumor sample cohort analyzed, all preservation techniques seem to have a similar degree of RNA integrity. **d)** UMAP projection of the cell clusters identified by integrating 6 tumor and 10 CSF samples together and colored by cell cluster initial annotations, prior to re-clustering. **e)** UMAP projection in (d) colored by sample preservation technique. It shows how there is no batch effect by sample preservation technique. **f)** UMAP projection in (d) colored by patient ID. It shows how there is no batch effect by patient sample. **g)** Heatmap representing the relative abundance of each cell type, measured as the proportion of cells of the cell type vs the total of sequenced cells, across CSF samples in the UMAP projection in Fig. 3c. Rows represent cell types, sorted by variance (represented on the left, standard deviation from low to high). Columns represent CSF samples, sorted according to the relative abundance of cytotoxic lymphocytes. Some cell types have been aggregated; TAMs 1 and 2 into TAMs cells and cytotoxic lymphocytes [>>Tcell] and [>>NK] into cytotoxic lymphocytes. Top panel shows a stacked bar plot representing the relative abundance of each cell type. A color annotation is shown in the right, matching the cell type colors in (b). **h)** Line plot representing the changes in the relative abundance of each cell type in g) in two patients (P6 and P9) where several time-point CSF samples were collected.

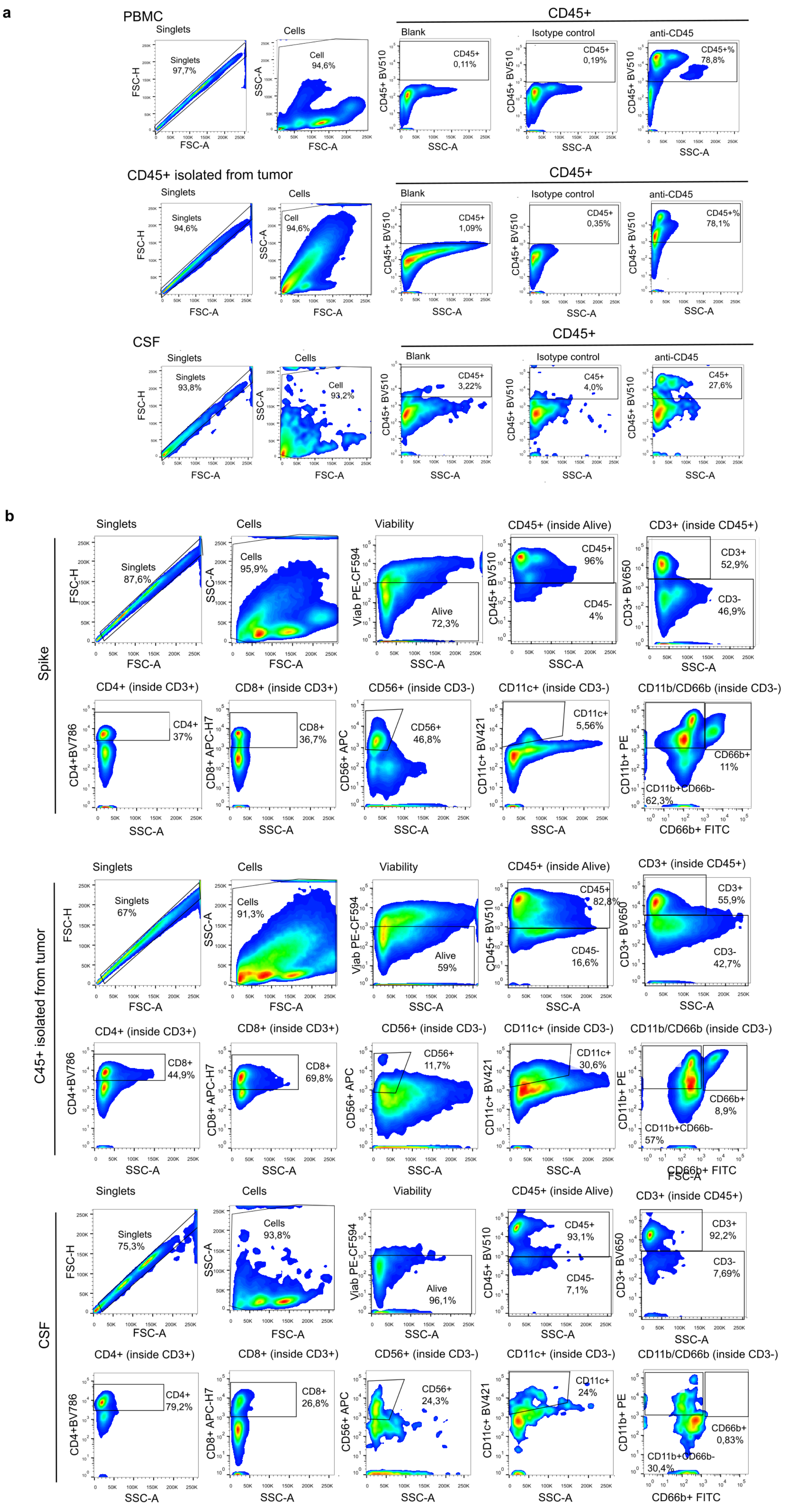
a
PBMC
Singlets
Singlets 97,7%
FSC-H
FSC-A
Cells
Cell 94,6%
SSC-A
CD45+
Blank
CD45+ 0,11%
CD45+ BV510
Isotype control
CD45+ 0,19%
anti-CD45
CD45+% 78,8%
CD45+ isolated from tumor
Singlets 94,6%
Cell 94,6%
CD45+ 1,09%
CD45+ 0,35%
CD45+% 78,1%
CSF
Singlets 93,8%
Cell 93,2%
CD45+ 3,22%
C45+ 4,0%
C45+ 27,6%
b
Spike
Singlets 87,6%
Cells 95,9%
Viability
Viab PE-CF594
Alive 72,3%
CD45+ (inside Alive)
CD45+ 96%
CD45- 4%
CD3+ (inside CD45+)
CD3+ BV650
CD3+ 52,9%
CD3- 46,9%
CD4+ (inside CD3+)
CD4+BV786
CD4+ 37%
CD8+ (inside CD3+)
CD8+ APC-H7
CD8+ 36,7%
CD56+ (inside CD3-)
CD56+ APC
CD56+ 46,8%
CD11c+ (inside CD3-)
CD11c+ BV421
CD11c+ 5,56%
CD11b/CD66b (inside CD3-)
CD11b+ PE
CD66b+ 11%
CD11b+CD66b- 62,3%
CD66b+ FITC
C45+ isolated from tumor
Singlets 67%
Cells 91,3%
Alive 59%
CD45+ 82,8%
CD45- 16,6%
CD3+ 55,9%
CD3- 42,7%
CD8+ 44,9%
CD8+ 69,8%
CD56+ 11,7%
CD11c+ 30,6%
CD66b+ 8,9%
CD11b+CD66b- 57%
CD66b+ FITC
FSC-A
CSF
Singlets 75,3%
Cells 93,8%
Alive 96,1%
CD45+ 93,1%
CD45- 7,1%
CD3+ 92,2%
CD3- 7,69%
CD4+ 79,2%
CD8+ 26,8%
CD56+ 24,3%
CD11c+ 24%
CD66b+ 0,83%
CD11b+CD66b- 30,4%

Supplementary figure 8, associated with Figure 3. Flow cytometry immune cell type identification through the general leukocyte panel

a) FC representative control plots of the indicated samples. Blank (no antibodies), isotype control (all the antibodies plus IgGk1-BV510), anti-CD45$^+$ (all the antibodies plus anti-CD45-BV510). These controls referred to panels displayed in (a). **b)** FC gating strategy of leukocyte population comparing control (tumor sample with a spike of PBMCs), representative CD45+ isolated cells from a tumor sample, and a representative CSF sample. CD45+ (total leukocyte), CD3$^+$ (T cells), CD8$^+$ (CD8$^+$T cells), CD4$^+$ (CD4$^+$T cells), CD56$^+$ (NK cells), CD19$^+$ (B cells), CD11b$^-$/CD66b$^+$ (Neutrophils), CD11c (DC) and CD11b$^+$/CD66b$^-$ (Macrophages, TAMs).

a

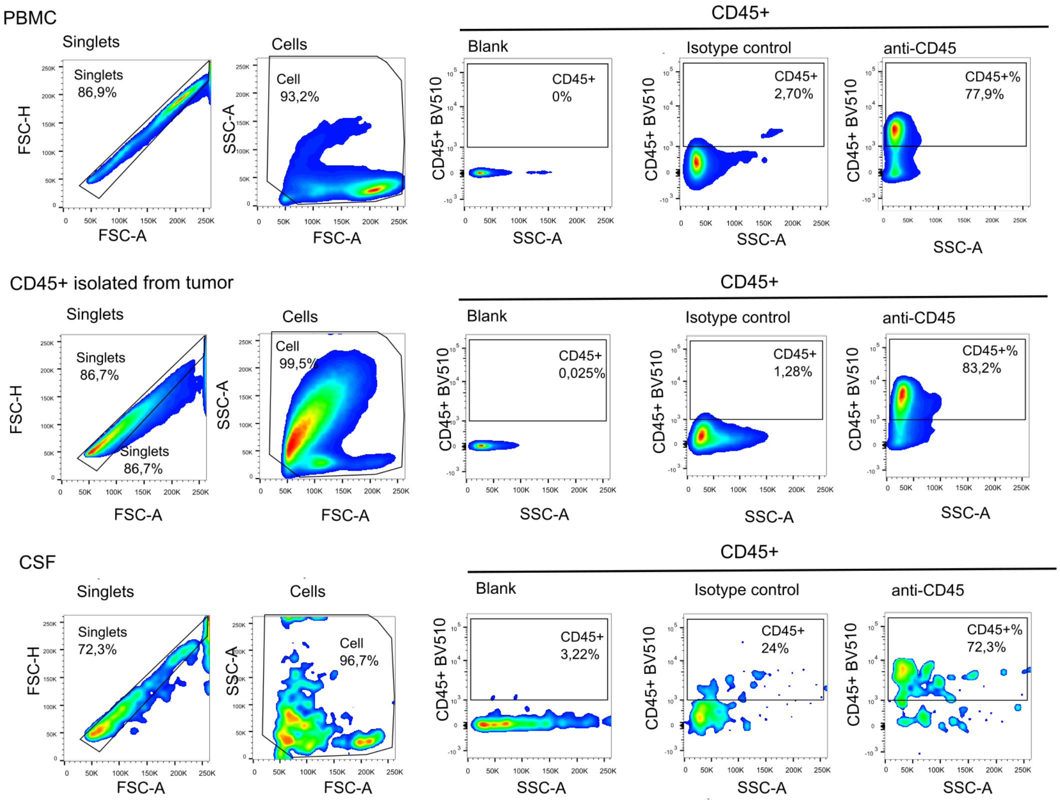
PBMC
Singlets
Singlets 86,9%
FSC-H
FSC-A
Cells
Cell 93,2%
SSC-A
FSC-A
CD45+
Blank
CD45+ 0%
CD45+ BV510
SSC-A
Isotype control
CD45+ 2,70%
CD45+ BV510
SSC-A
anti-CD45
CD45+% 77,9%
CD45+ BV510
SSC-A
CD45+ isolated from tumor
Singlets
Singlets 86,7%
Singlets 86,7%
FSC-H
FSC-A
Cells
Cell 99,5%
SSC-A
FSC-A
CD45+
Blank
CD45+ 0,025%
CD45+ BV510
SSC-A
Isotype control
CD45+ 1,28%
CD45+ BV510
SSC-A
anti-CD45
CD45+% 83,2%
CD45+ BV510
SSC-A
CSF
Singlets
Singlets 72,3%
FSC-H
FSC-A
Cells
Cell 96,7%
SSC-A
FSC-A
CD45+
Blank
CD45+ 3,22%
CD45+ BV510
SSC-A
Isotype control
CD45+ 24%
CD45+ BV510
SSC-A
anti-CD45
CD45+% 72,3%
CD45+ BV510
SSC-A

b

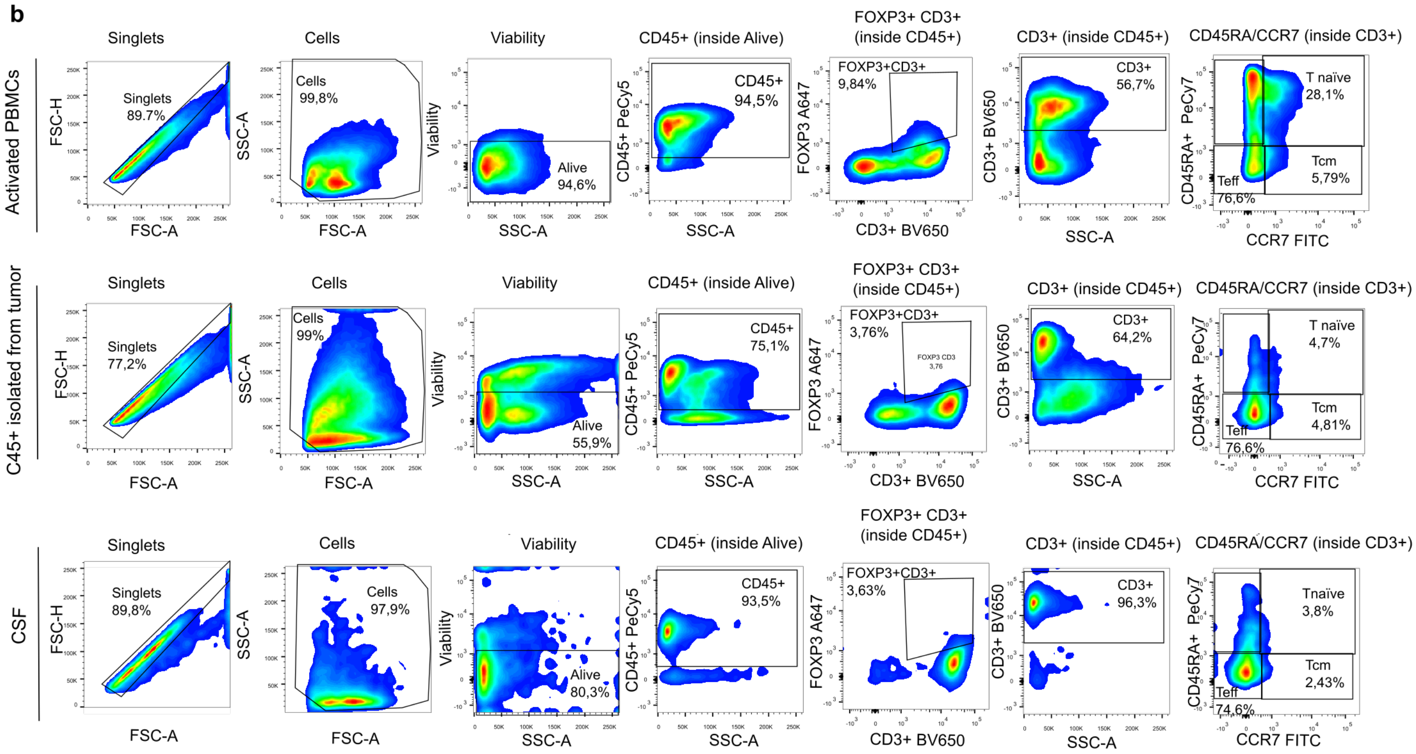
Activated PBMCs
Singlets
Singlets 89.7%
FSC-H
FSC-A
Cells
Cells 99,8%
SSC-A
FSC-A
Viability
Alive 94,6%
Viability
SSC-A
CD45+ (inside Alive)
CD45+ 94,5%
CD45+ PeCy5
SSC-A
FOXP3+ CD3+ (inside CD45+)
FOXP3+CD3+ 9,84%
FOXP3 A647
CD3+ BV650
CD3+ (inside CD45+)
CD3+ 56,7%
CD3+ BV650
SSC-A
CD45RA/CCR7 (inside CD3+)
T naïve 28,1%
Teff 76,6%
Tcm 5,79%
CD45RA+ PeCy7
CCR7 FITC
C45+ isolated from tumor
Singlets
Singlets 77,2%
FSC-H
FSC-A
Cells
Cells 99%
SSC-A
FSC-A
Viability
Alive 55,9%
Viability
SSC-A
CD45+ (inside Alive)
CD45+ 75,1%
CD45+ PeCy5
SSC-A
FOXP3+ CD3+ (inside CD45+)
FOXP3+CD3+ 3,76%
FOXP3 A647
CD3+ BV650
CD3+ (inside CD45+)
CD3+ 64,2%
CD3+ BV650
SSC-A
CD45RA/CCR7 (inside CD3+)
T naïve 4,7%
Teff 76,6%
Tcm 4,81%
CD45RA+ PeCy7
CCR7 FITC
CSF
Singlets
Singlets 89,8%
FSC-H
FSC-A
Cells
Cells 97,9%
SSC-A
FSC-A
Viability
Alive 80,3%
Viability
SSC-A
CD45+ (inside Alive)
CD45+ 93,5%
CD45+ PeCy5
SSC-A
FOXP3+ CD3+ (inside CD45+)
FOXP3+CD3+ 3,63%
FOXP3 A647
CD3+ BV650
CD3+ (inside CD45+)
CD3+ 96,3%
CD3+ BV650
SSC-A
CD45RA/CCR7 (inside CD3+)
Tnaïve 3,8%
Teff 74,6%
Tcm 2,43%
CD45RA+ PeCy7
CCR7 FITC

c

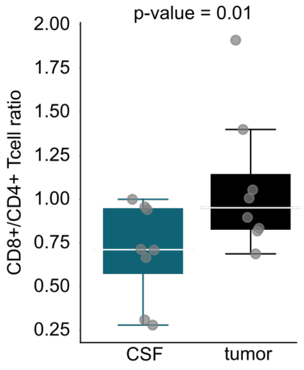
p-value = 0.01
CD8+/CD4+ Tcell ratio
2.00
1.75
1.50
1.25
1.00
0.75
0.50
0.25
CSF
tumor

Supplementary figure 9, associated with Figure 3. Flow cytometry immune cell type identification through the T cell panel

a) FC representative control plots of the indicated samples. Blank (no antibodies), isotype control (all the antibodies plus IgGk1-PeCy5), anti-CD45$^+$ (all antibodies plus anti-CD45-PeCy5). These controls referred to FC panels displayed in (I). **b)** FC gating strategy of T cell subpopulation comparing control (activated PBMC with coated anti-CD3 and soluble anti-CD28 for 72h), a representative CD45$^+$ isolated cells from tumor sample, and a representative CSF sample. CD45$^+$ (total leukocyte), CD3$^+$ (T cells), FOXP3$^+$/CD3$^+$ (Treg), CCR7$^+$/CD45RA$^+$ (T naïve), CCR7$^-$/CD45RA$^-$ (T effector memory) , CCR7$^+$/CD45RA$^-$ (Tcm, T central memory). **c)** Box plot representation of the CD8/CD4$^+$ T cell ratio in the tumor vs the CSF in the FC sample cohort (n=8 patients, n=16 samples). The significance was assessed through the Wilcoxon test. All boxplots indicate median (center line), 25th and 75th percentiles (bounds of box), and minimum and maximum (whiskers).

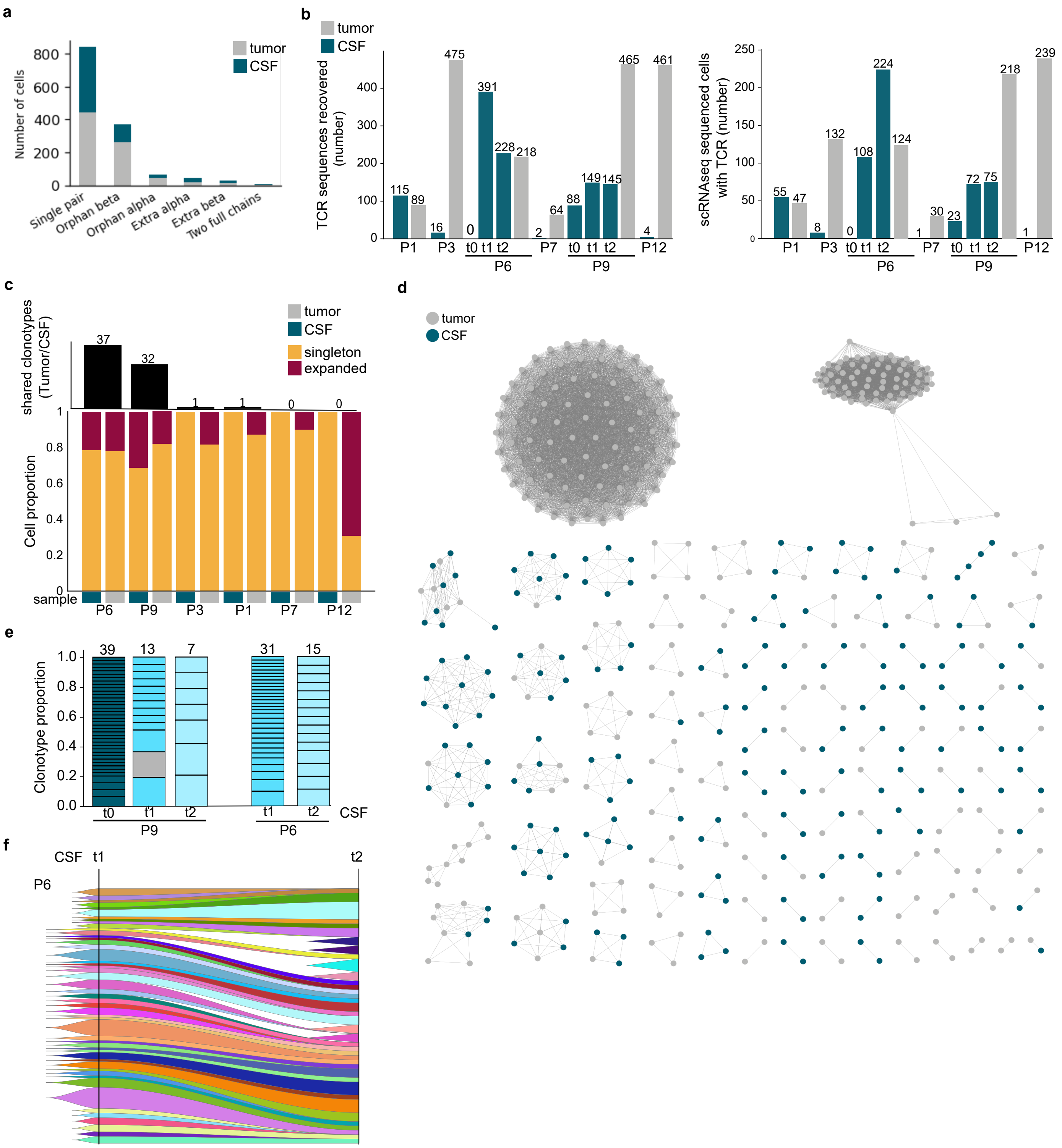

a
Number of cells
tumor
CSF
Single pair
Orphan beta
Orphan alpha
Extra alpha
Extra beta
Two full chains
b
TCR sequences recovered (number)
scRNAseq sequenced cells with TCR (number)
P1
P3
P6
P7
P9
P12
c
shared clonotypes (Tumor/CSF)
Cell proportion
singleton
expanded
sample
d
e
Clonotype proportion
f
CSF
t1
t2

Supplementary figure 10, associated with Figure 4. T cell TCR profiling across BrM.

a) Stacked plot representing the distribution of reads recovered from the distinct TCR chains (n=6 patients, 15 samples). **b)** Left panel, bar plot representing the number of TCR sequences recovered by patient sample. Right panel, bar plot representing the number of cells sequenced by scRNAseq where a TCR could be sequenced. **c)** Distribution of TCR clonotypes per patient sample. Top panel; bar plot representing the number of shared TCR sequences between matched tumor-CSF samples of the same patient. Bottom panel, stacked bar plot representing the proportion of expanded/single TCR clonotypes per patient sample. **d)** Network representation of TCR clonotypes, nodes represent cells and have been connected when sharing TCR sequences. Nodes are colored according to the cell of origin, tumoral or CSF. **e)** Stacked bar plot representing the abundance of each expanded TCR clonotype, in multiple time point CSF samples from two patients (P6 and P9). **f)** Fish plot representing the clonal evolution of CSF TCR sequences in the two distinct time points in patient P6.
