# Supplementary material for: Immune cell profiling of the cerebrospinal fluid enables the characterization of the brain metastasis microenvironment
Source: Nat Commun. 2021 Mar 8;12:1503. doi: 10.1038/s41467-021-21789-x (PMC7940606; doi:10.1038/s41467-021-21789-x)
Supplement: Supplementary file 3 — Description of Additional Supplementary Files [file 41467_2021_21789_MOESM3_ESM.pdf]

Description of Additional Supplementary Files

File Name: Supplementary Data 1
Description: Patient annotations table including: experiments performed with each patient sample and clinical annotations.

File Name: Supplementary Data 2
Description: Matrix of targeted gene expressions through PanCancer Immune panel v.1 containing all normalised gene counts.

File Name: Supplementary Data 3
Description: Enrichment scores of seven immune populations and IFNɣ signature for 48 BrM samples and the immune cluster (as shown in Fig. 1c).

File Name: Supplementary Data 4
Description: Whole exome sequencing output annotated by the Cancer Genome Interpreter for 6 patients; including all the mutations in genomic and protein coordinates, as well as the driverness annotation.

File Name: Supplementary Data 5
Description: CD8+ T cell IHC quantification data.

File Name: Supplementary Data 6
Description: Flow cytometry data corresponding to general immune cell population and T cells subpopulations percentages in tumor and CSF samples.

File Name: Supplementary Data 7
Description: scRNAseq cell annotations table from tumor cohort analysis (associated to Fig. 2; n=9 patients, 9 samples) including: patient ID, sample type, cell ID, cell cluster, cell cycle score, TCR clonotype classification.

File Name: Supplementary Data 8
Description: scRNAseq cell annotations from matched tumor-CSF cohort analysis (associated to Fig. 3; n=6 patients, 16 samples) including: patient ID, sample type, cell ID, cell cluster, cell cycle score, TCR clonotype classification.

File Name: Supplementary Data 9
Description: TCR sequences per cell, including alpha and beta chains; from the tumor-CSF cohort analysis (associated to Fig.4; n=6 patients, 15 samples).
